# Governance in clinical academic medical school departments: A time for change

**DOI:** 10.1002/acm2.13767

**Published:** 2022-08-23

**Authors:** Timothy D. Solberg

**Affiliations:** ^1^ Department of Radiation Oncology University of Washington Seattle Washington USA

**Keywords:** governance, leadership, recruiting, term‐limits

Throughout my career, I've often found myself perplexed by how academic medical centers (AMCs) are managed. I'm also not so naïve as to think that challenges are unique to AMCs; early in my life I held a management position with a Fortune 500 company, and more recently I held a career position within the federal government. Indeed, the public sector, private industry, and AMCs can all exhibit poor decision making, communication challenges, reluctance toward change and innovation, tunnel vision, risk aversion, and so on. That said, there are unique aspects in academic health care, including highly intelligent and well compensated individuals working in a matrixed organizational structure in which the fundamental tripartite mission of teaching, research, and clinical care are often at odds.

In a series of interviews of faculty at five US medical schools, Pololi et al. found “… an intensely individualistic and competitive environment where rewards are usually accorded to individual contributions.^1^ Respondents perceived that individuals and institutions tend to function on behalf of their own self interests.”^1^ Faculty also reported “… a significant lack of alignment between their own and perceived institutional values. In particular, numerous faculty perceived a lack of attention to the social mission of providing care for all people and to the community, a lack of prioritization of excellence in clinical care, a devaluing of educational roles, questionable ethical behavior among leadership or management, and the necessity for self‐promoting behavior to achieve success.”^2^ There was also a sense of prioritizing publications, grants, and speaking engagements, to the detriment of commitment to care. Krupat et al. went on to describe leadership in AMCs as “… hierarchical, unwelcoming of differences, and nontransparent,” in a culture that “discourages humanistic orientations, and those who seek professional rewards learn that the preferred route is through competition and self‐promotion rather than collaboration.^3^” And James Schroetlin has described the model under which academic medicine operates as “feudal.”^4^

There are a number of fundamental practices that contribute to challenges in academic medicine, although for this editorial I will focus only on two, appointing/promoting from within, and lack of term limits. Additionally, I largely limit my observations to those regarding chairs of clinical departments. Admittedly my dataset is incomplete, consisting of the six institutions for which I've worked, plus discussions with colleagues from a number of other AMCs. As a frame of reference, I've spent my career in academic medicine and had significant opportunity to observe the operations and institutional politics of AMC, and notably, that of a number of deans, C‐suite administrators, and department chairs. I've personally worked for eight chairs and two interim chairs of radiation oncology departments. I've developed both professional and personal relationships with radiation oncology chairs at other institutions, as well as chairs of other departments including neurosurgery, radiology, urology, and surgery. And I have participated on chair search committees on multiple occasions.

A “national search,” overseen by a search committee composed of other chairs and senior faculty and administrators, is the standard mechanism for recruiting for open leadership positions. While unsolicited applications are generally accepted, potential candidates are often identified by the committee, and more recently, complemented by executive placement firms. A primary mission of the committee is to identify and recommend qualified candidates, given institutional goals and priorities outlined by a dean.

Despite the pretense of a “national search,” many seem to be predetermined. Certainly, we all know individuals who were appointed from within their own institution. While many internal candidates are indeed the most qualified, there are also those appointed for reasons other than aptitude and merit, for example, concerns that sought‐after faculty may be lured elsewhere, to expedite the search process or to save on the recruiting package. At one of the institutions for which I worked, only six of 28 clinical chairs were appointed from outside the institution. Statistically it is not possible that the 22 recruited from within were truly the best candidates available nationally, so one must assume that processes such as these were engineered, at least in part. Ultimately, routine appointing from within can erode confidence in the fairness and equity of the process, both within and outside the institution. More importantly, appointing from within degrades diversity of culture, experience, and thought, and in the long term, is actually detrimental to the institution, leading to underachievement and stagnation. And individuals appointed thusly may be viewed as less qualified or compromised, whether such a label is valid or not.

Shortly after I began graduate studies at UC Davis, there was a change in leadership in the Physics Department. Curious, I inquired about the process and was informed that the chair changed every 3 years, and that it was an obligation that most faculty were expected to take on at some point in their tenure. The role was largely one of faculty advocacy, and in exchange for the burden of administrative responsibilities, there was a somewhat lighter teaching load. While this practice is common in the sciences and in the humanities, the contrast within AMCs is significant. There are reports of individuals serving in a chair role for 25–40 years or more, and for each report there are likely dozens more.^5^, ^6^, ^7^, ^8^ Fisher observed that “…the perks of the position, the sense of entitlement, the stipend, and the (superficial) deference can prove to be addictive.”^9^ Beeler et al. noted that “… it has become atypical for department chairs or division chiefs to leave their posts unless they assume a higher leadership position, a pattern that has resulted in terms that are far longer than is necessary to preserve continuity or institutional memory.”^7^ They further remarked that this “… results in a leadership model in which one person's perspective holds disproportionate sway, sometimes for decades.”^7^ Austin asserted that “… no mechanism short of death or retirement exists to transfer power from the individuals in these leadership positions,” that these individuals become “experienced non‐experts,” and that “…passionate, visionary, and/or transformative leaders … are rare.”^8^

Recently, there has been a renewed call for real term limits in academic medicine. Austin has pointed out the many advantages of term limits and proposed a maximum of two renewable 4‐year terms for AMC division heads, department heads, deans, and presidents.^8^ Beeler et al. accentuated the positive impact that term limits have on diversity, equity, and inclusion.^7^ It is interesting to note that at Mayo Clinic, routinely ranked the number one health system in the United States, chairs rotate every 8 years, and with minimal changes to compensation based on leadership roles and no recruiting packages.^8^ Clearly Mayo is on to something.

In summary, I would echo recommendations that AMCs adopt rational term limits for leadership positions, and ensure honest, objective national searches. Additional measures that can help to ensure a culture of transparency, respect, and professionalism include:

1. Insist that leaders govern fairly and transparently, seeking consensus when possible;
2. Promote a holistic management approach, connecting every individual in the enterprise, inviting input, and minimizing one‐on‐one exchanges;
3. Formalize leadership training in self‐awareness, hubris management, effective communication skills, and trust building^10^;
4. Perform annual reviews of senior leaders, utilizing a 360 process to ensure input from all groups, and importantly, providing appropriate institutional feedback following each review;
5. Develop working strategic plans, with tangible goals and timelines, shared institutionally and re‐evaluated regularly;
6. Provide leaders authority commensurate with their responsibility;
7. Develop clear policies and procedures, and ensuring that they are applied equitably;
8. Provide for centralized, transparent financial accounting throughout the enterprise;
9. Incentivize leaders to maintain their clinical roles and avoid disconnecting from clinical medicine^8^;
10. And provide mechanisms for those termed‐out individuals to exit leadership positions gracefully as well as predictably^8^.

As a closing comment, I believe many of the salient points and recommendations also apply to those who lead academic physics divisions. If my own career path is any indication, I have regularly followed my own advice.

## CONFLICT OF INTEREST

The author declares that there is no conflict of interest that could be perceived as prejudicing the impartiality of the research reported.
